# Factors shaping good and poor nurse-client relationships in maternal and child care: a qualitative study in rural Tanzania

**DOI:** 10.1186/s12912-022-01021-x

**Published:** 2022-09-05

**Authors:** Kahabi Isangula, Eunice S. Pallangyo, Columba Mbekenga, Eunice Ndirangu-Mugo, Constance Shumba

**Affiliations:** 1 School of Nursing and Midwifery, The Aga Khan University, Dar Es Salaam, Tanzania; 2 School of Nursing and Midwifery, The Aga Khan University, Nairobi, Kenya

**Keywords:** Maternal and Child Care, Nurse-client relationship, Patient-provider relationship, Rural, Tanzania, Africa

## Abstract

**Background:**

Evidence indicates that poor nurse-client relationships within maternal and child health (MCH) continues to impact trust in formal healthcare systems, service uptake, continuity with care and MCH outcomes. This necessitates contextualized innovative solutions that places both nurses and clients at the forefront as agents of change in optimizing intervention designs and implementation. This study explored nurses and clients’ perspectives on the factors shaping nurse-client relationships in MCH care to generate evidence to guide subsequent steps of human centered design (HCD) that involve designing effective strategies for improving therapeutic relationships in Shinyanga, Tanzania.

**Methods:**

Qualitative descriptive design was employed. About 9 Focus Group Discussions (FGDs) and 12 Key Informant Interviews (KIIs) with purposefully selected nurses and midwives, women attending MCH services and administrators were conducted using semi-structured interview guides in Swahili language. Data were transcribed and translated simultaneously, managed using Nvivo Software and analyzed thematically.

**Results:**

Factors shaping nurse-client relationships were heuristically categorized into nurse, client and health system factors. Nurse contributors of poor relationship ranged from poor reception and hospitality, not expressing care and concern, poor communication and negative attitudes, poor quality of services, job dissatisfaction and unstable mental health. Client contributors of poor relationship include being ‘much know’, late attendance, non-adherence to procedures and instructions, negative attitudes, poor communication, inadequate education and awareness, poverty, dissatisfaction with care, faith in traditional healers and unstable mental health. Health system contributors were inadequate resources, poor management practices, inadequate policy implementation and absence of an independent department or agency for gathering and management of complaints. Suggestions for improving nurse-client relationship included awards and recognition of good nurses, improving complaints mechanisms, continued professional development, peer to peer learning and mentorship, education and sensitization to clients, improving service quality and working conditions, improving renumeration and incentives, strengthening nursing school’s student screening and nursing curriculum and improving mental health for both nurses and clients.

**Conclusions:**

The factors shaping poor nurse- client relationships appear to extend beyond nurses to both clients and healthcare facilities and system. Implementation of effective interventions for addressing identified factors considering feasibility and acceptance to both nurses and clients using novel strategies such as HCD could pave the way for employing good nurse-client relationships as a tool for improving performance indicators and health outcomes within MCH care.

**Supplementary Information:**

The online version contains supplementary material available at 10.1186/s12912-022-01021-x.

## Background

Nurses and midwives form a critical component of maternal and child health (MCH) services globally. They play a vital role in the delivery of primary health-care services related to pregnancy monitoring, delivery and postnatal care for women and newborns around the world [1, 2, –4]. In sub-Saharan Africa, nurses and midwives are often respected members of the community and provide advice and evidence-based information on a range of health issues, including care of newborns and young children [2–4]. In the presence of sufficient, well supported, and competent nurses and midwives, 83 per cent of maternal deaths, stillbirths and neonatal deaths could be prevented [5–7]. It is considered that competent nurses and midwives have the potential to increase client service uptake, continuity, and consequently improve health outcomes, such as increased breastfeeding initiation and duration, reductions in caesarean sections, maternal infections, postpartum hemorrhage, and preterm births [8]. Literature suggests that investing in nurses and midwives has the potential to yield a 1,600 per cent (16-fold) return on investment resulting from improved MCH outcomes [9].

Despite their critical role, there has been increasing clients’ dissatisfaction with nurses and midwives’ interpersonal and perceived technical competences within MCH care in the recent years [10–19]. Perceived technical incompetence associated with skills, reliability, assurance, confidentiality, and patient engagement and, behavioral incompetence involving demeanor/attitudes empathy, communication skills/language, violation of client rights and respect, continue to obscure the positive value of nurses and midwives in the delivery of MCH interventions in Tanzania and elsewhere [10–21]. Recent studies indicate that clients’ dissatisfaction with nurses’ interpersonal and technical aspect of care continues to erode client trust in formal healthcare system, service uptake, continuity and MCH outcomes [19–23].

To address clients’ dissatisfaction, healthcare service governance instruments including policies, client service charters, health facility governance committees, complaints mechanisms and professional bodies have been emphasized in both high- and low-income settings but their effectiveness is not well-established. Consequently, political interventions such as employment termination remain the cornerstone of addressing this complex problem which further creates tension between clients and the politically pronounced ‘bad, lazy and incompetent’ nurses contributing to loss of providers’ morale [24, 25]. Competence based interventions focusing on provider communication skills and patient centered care and, patient literacy, information seeking, participation and questioning skills are often implemented on adhoc basis yielding unsatisfactory results. A major challenge with existing interventions documented in literature is the failure to address all the complexities of nurse-client relationships along the continuum of MCH care. Patient’s socio-economic fragility, literacy, and behaviours as well as providers’ competence and behaviours, and health system challenges particularly in rural contexts add to the complexity of nurse-client relationships. Rurality adds to the complexity of nurse-client relationships partly because of limited healthcare resources and infrastructures and the prevailing competition between informal and traditional systems which continue to impact uptake and continuity of formal MCH services [15, 23].This complexity necessitates targeted contextualized innovative solutions that places nurses and clients at the forefront as agents of change in optimizing intervention designs and implementation [26–28]. A human centered design (HCD) is one of the innovative approaches to problem-solving that leverages insights from the end-users of services, and experiences in order to develop best-fit solutions that are rapidly prototyped and iteratively refined [26–32]. HCD is considered to facilitate improvements in client, provider, and community satisfaction, as well as increased efficiency and collaboration in public health intervention development and implementation process [30–32]. Furthermore, HCD may result in more successful and sustainable interventions compared to traditional problem-solving approaches in health care and public health [31]. HCD approach also embraces a system-wide outlook by considering interactions of factors at different levels and harmonizing individual interests to form collective interests when developing solutions [30–32]. The aim of this study was to explore nurses and clients’ perspectives of factors shaping nurse-client relationships in MCH care. The findings of the study are expected to generate evidence to guide subsequent steps of HCD process that involve designing effective strategies for improving nurse-client relationships in Shinyanga, Tanzania.

## Methods

### Design

The HCD protocol with a description of this inquiry has been recently published elsewhere [33]. Briefly, a Qualitative descriptive study design was employed. Qualitative descriptive design was selected because is a well-accepted design within the qualitative domain for answering the questions of ‘what’ are the drivers of poor-nurse client relationships in MCH care [34]. Although a mixed method and/or a theory-driven inquiry was also considered, it was deemed impractical for this inquiry because a focus was on generating rich perspectives on nurse-client relationship to guide further HCD steps [33].

### Settings

This study was conducted in Shinyanga, a region located in the Lake Zone and predominantly inhabited by Bantus. Isangula [23] offers a detailed description of the region. Briefly, Shinyanga falls within the low-income category of the regions in Tanzania. It is administratively divided into five districts: Shinyanga Municipal Council (MC), Shinyanga District Council (DC), Kishapu DC, Kahama MC and Kahama DC. The choice of Shinyanga Region is twofold. First, the region ethnically, is predominantly inhabited by Sukuma who share a range of socio-cultural beliefs and practices with minimal diversity. Due to its near homogeneity, the region is a perfect exemplar of many other rural regions of Tanzania. Second, despite a number of capacity building interventions, local data indicate enormous concerns of poor provider–client relationships in MCH care [23]. Within Shinyanga Region, Shinyanga MC was purposefully selected because patients in this district have wealthier access to both the formal healthcare system (mostly public and few private and faith-based facilities) and traditional care [23].

### Study population, sample size and sampling method

A total of 9 FGDs with purposefully selected nurses and MCH clients and 12 KIIs with MCH stakeholders were conducted. While equal representation is not a primary focus in qualitative studies [34], the level and ownership of facility (public, private & faith based and; dispensary, health center, and district hospital) were considered during participants’ enrollment. No age preference was made for this qualitative inquiry other than being a nurse/midwife who has been working in MCH for at least two years OR a client currently attending to MCH clinics and has made at least three visits in a year OR MCH service administrator in Shinyanga.

### Recruitment of participants

Recruitment for FGDs participants commenced by purposeful selection of healthcare facilities considering ownership, level and availability of MCH services. Then, a courtesy visit was made to Shinyanga district medical officer for approval to visit the facilities. This was followed by a physical introductory visit to the facilities where the study information was provided to the healthcare facility incharge. The facility incharge assisted with identification of one provider with good interpersonal communication skills within the selected facility to serve as an ‘enrolment assistant’. Each proposed enrolment assistant was briefly oriented on the study to facilitate recruitment of providers and MCH clients and was excluded from FGDs. The enrolment assistant shared information about the study during clinical meetings (for recruitment of providers) and MCH visits (for recruitment of clients) and registered those who expressed interest. This was followed by subsequent visits by research assistants for scheduling and conducting interviews. Recruitment for KII was done by initial phone contact with MCH administrators after obtaining the phone numbers from the district medical office. During phone calls with administrators, study information was offered, request to participate made and interviews scheduled with those agreeing to take part in the study considering their preferences of place and date.

### Data collection tools

Semi-structured FGDs and KII guides were developed and translated through a consultative process involving experts at the Aga Khan University. The English versions of interview guides were translated into Swahili language then back translated to English and checked for conceptual equivalence. Questions within the interview guide ranged from those examining experiences of poor and good nurse -client relationships, contributors of poor nurse -client relationships to those examining suggestions and considerations for improving nurse-client relationships.

To ensure collection of rich data, three research assistants who are native Swahili speakers with Diploma in medical and social sciences were recruited and trained on the use of interview guides and techniques pertaining to this study. The interview guides were pre-tested in purposefully selected settings. After pre-testing, the guides were refined by rephrasing some of the questions and adding more probes to ensure readiness for use in the actual data collection process. Close and supportive supervision of research assistants was conducted throughout data collection and analysis stages to ensure data quality.

### Data collection

Interviews were conducted in a place and date preferred by the participants. Before commencement of FGDs and KIIs, participants were given information about the study, risk and benefits of participation (information sheet was part of the interviews). A verbal consent for interview and voice recording were sought in advance. Then, interview sessions lasting for approximately 30–60 min were conducted in a safe environment. In view of recent Covid-19 pandemic, we limited the number of FGD participants to between 6 and 8 depending on the size of the venue to ensure that adequate social distance is maintained. Likewise, all participants and research assistants were provided with face masks and hand sanitizers throughout the interviews.

### Data management and analysis

FGDs and KIIs data transcription and translation occurred simultaneously by research assistants and verified by the research team. After transcription and translation, all interview transcripts were de-identified, pseudonyms generated for each participant and the data uploaded into NVivo 12 software (QSR International, Australia) for management and deductive thematic coding. Qualitative data analysis employed thematic analysis strategy described by Braun & Clarke [35]. A stepwise approach was used for a deductive thematic analysis of the interview transcripts. To begin with, first, KI examined the research questions and generated several initial themes and subthemes. Then, these themes and subthemes were subjected to review by the research team (ES, CN & EN-M) generating a consensual list. This resulted into an analytical matrix of the main themes and subthemes which then transcended as codes and subcodes respectively during coding. Then, KI exported individual transcripts and phrases representing participants’ responses to investigators’ probes to relevant themes and related subthemes within Nvivo Software. KI continued coding the transcripts and collated all relevant coded data extracts within pre-identified themes. Throughout coding, the peer consultation was maintained to reflect on the themes and related data extracts. A consensus based approach was used to resolve emerging disagreements as well as making a decision on whether to include codes that do not fit within the pre-developed themes and subthemes or disbandment when subjectively and objectively deemed of no critical value to the study. Then, the data within the Nvivo were exported to Ms Word for interpretation and report generation.

## Results

### Participant’s demographics

The study involved 12 KIIs with MCH administrators and stakeholders and, 9 FGDs with 66 nurses and MCH clients (4 FGDs with 30 nurses and 5 FGDs with 36 MCH clients). On the one hand, majority of KII participants were Female (67%), aged between 40–49 years (67%) with university education (75%) and more than 5 years of experience in MCH leadership (75%). On the other hand, considering all 66 FGD participants, majority were female (94%), aged less than 39 years (83%) and married. Most MCH clients had primary education and 1–2 children with most nurses having college/university education (Table 1).

**Table 1:** Participants demographics

**PARTICIPANT DEMOGRAPHIC**				
**KII**	**(*n* = 12)**	**Total (%)**		
**Gender**	Male	4 (33)		
	Female	8 (67)		
**Age**	< 30	0		
	30–39	3 (25)		
	40–49	8 (67)		
	> 50	1 (8)		
**Education**	Secondary	1 (8)		
	College	2 (17)		
	University	9 (75)		
**Years of Leadership in MCH**	< 2	1 (8)		
	2–4	2 (17)		
	> 5	9 (75)		
**FGD**	***N* = 66, 9FGDs**	**CLIENTS (*n* = 36, 5FGDs)**	**NURSES (*n* = 30, 4 FGDs)**	**Total (%)**
**Gender**	Male	0	4	4 (6)
	Female	36	26	62 (94)
**Age**	< 30	22	6	28 (42%)
	30–39	13	14	27 (41%)
	40–49	0	5	5 (8%)
	> 50	1	5	6 (9%)
**Marital status**	Single	8	3	11
	Married	28	27	55
**Education**	None	5	0	5
	Primary	17	1	28
	Secondary	9	6	15
	College/University	5	23	28
**Number of children**	1–2	21		
	3–4	8		
	> 5	7		

### Factors shaping poor nurse-client relationship

Participants were asked to describe the contributors of poor nurse-client relationship in this rural Tanzanian contexts. Looking across transcripts, the contributors of poor nurse-client relationship were heuristically categorized into three groups: Nurse contributors, client contributors and healthcare system contributors. It is important to note that there was a tendency among most nurses to post blames to clients on the one hand and, clients posting blames to nurses on the other hand. Although Table 2 offers more details, each of these contributors are described below.

**Table 2:** Contributors to poor nurse-client relationships

**Nurse contributors**	**Client contributors**	**Healthcare sector contributors**
**Poor reception and hospitality** • *Negative reception* • *Self-pride among nurses* • *Not greetings clients* • *Not responding to greetings*	**The ‘much know’ client** • *Clients who know everything-medicine, how to give injection *etc • *Clients basing their expectations on information from internet sources*	**Inadequate resources** • *Inadequate/shortage of nurses and other providers amidst high client loads* • *Inadequate medicines and medical supplies* • *Inadequate healthcare infrastructure* • *Dysfunctional service delivery HIS e.g., GoTHOMIS not sending patient information to departments timely* • *Non-dissemination of guidelines and SOPs to facilities*
	**Delayed clinic attendance/coming outside scheduled clinic hours (without emergency)** **Failure to adhere to established procedures for receiving care** **Harboring negative attitudes towards nurses** • *Negativity towards providers* • *Having negative information about nurses before facility visit* • *Having a negative experience with nurses in a similar or different facility* • *Believing that no better healthcare service without bride* • *Holding nurses in contempt*	
**Not expressing care and concern** • *Not conducting triage* • *Acting busy and ignoring patients* • *Doing personal activities instead of offering care (exchanging stories among fellows, chatting or preoccupation with phones)* • *Inadequate preparation for offering health care services*		
		**Poor human resource for health management practices** • *Bullying and mistreatment of nurses by administrators and leaders* • *Inadequate financial incentives ad motivations* • *Small and delayed salaries* • *Delayed promotions and salary increments*
**Poor communication** • *Bad and harsh language* • *Speaking with anger, shouting and verbal reprimands towards clients* • *Being or appearing naturally angry and troublesome* • *Not explaining things to clients clearly* • *Not listening to clients* • *Not making eye contacts when speaking* • *Not being able to speak local language (Sukuma)* • *Not setting adequate time to speak to clients* • *Lack of customer care skills*		
	**Poor communication** • *Being troublesome/ with bad language towards nurses* • *Having self-pride and disrespect towards nurses* • *Portraying anger when explaining problems* • *Being naturally non-civilized and angry because of cultural upbringing from childhood* • *Not receiving information early about absence of a certainservice*	**Lack of client trust towards facilities and healthcare providers** • *Bad reputation of the healthcare facility among community members* • *Bad reputation of nurses in the community e.g., physical abuse of patients* • *Negative attitudes of community members towards nurses* • *Inadequate orientation of new employees on nurse-client relationship*
		**Inadequate policy implementation** • *Delay in fund disbursement from central government for medicine and medical equipment which creates deficits that fuel client distrust towards nurses* • *Inadequate screening of nursing students in health training institutions leading to enrollment of those without nursing calling* • *Nonadherence to labor laws e.g., required working hours*
**Negative attitudes towards clients** • *Thinking that clients are instructing or teaching them what to do when explaining what services/treatment they would like to receive* • *Nurses using phrases/language that may be perceived as humiliating/shaming e.g., ‘you are giving birth every year without resting’’*	**Inadequate education, awareness, and poverty** • *Limited understanding among community members on health issues and process of care* • *Limited understanding of instructions* • *Nonadherence to instructions* • *Poor preparation before delivery* • *Failure to acknowledge many responsibilities nurses have* • *Non ownership of health insurance*	
		**Politicization of medicine for instance telling people that they would receive free care while no resources made available to fulfill such commitments** **High cost of care fueling complaints and dissatisfaction** **Ineffective complaints mechanism** • *Dysfunctional suggestion box system* • *Absence of an independent department or agency specifically responsible for gathering, analyzing, and communicating clients and providers complaints* • **Absence of specific individuals/agency for continued capacity building and mentorship of nurses on provider–client relationships** • **Inadequate mental health support for both nurses and clients**
**Poor relationship among nurses for instance nurse discrediting fellows to patients** **Job dissatisfaction** • *Not being satisfied with nursing job (i.e., income, working tools and transport)* • *Low work morale because of delated promotions or low income* • *Not meeting personal life goals as expected* • *Lack of ‘nursing calling’*		
	**Hurrying/forcing to receive certain services** • *Lack of patience and wanting to receive care ahead of others who came early* • *Using social status or position to force faster treatment/care* • *Forcing to receive certain services that they do not deserve or contrary to nursing profession guides*	
**Poor quality of services** • *Inadequate technical competence on certain services (therefore become harsh as a defensive mechanisms)* • *Offering substandard and poor care* • *Offering care in a hurry* • *Not offering appropriate education about side effects* • *Not performing one’s duties effectively* • *Extreme tiredness because of high workload and multiple shifts* • *Multiple responsibilities in different departments*		
	**Dissatisfaction with care** • *Coming with personal desires and expectations e.g., a nurse to receive care from or medications (lack of choices?)* • *Dissatisfaction with care when desires and expectations are not met*	
	**Trust in traditional healers and traditional birth attendants than in formal healthcare** **Unstable mental health** • *Mental health problems resulting from stresses of life* • *Loss of hope because of prolonged suffering from a disease*	
**Unstable mental health** • *Mental health problems resulting from stresses of life* • *Inadequate mental health support*		

#### Nurse contributors to poor nurse-client relationships

A number of factors emerged in participants’ accounts regarding nurse contributors of poor nurse-client relationships. Broadly, nurse contributors cited are mainly related to their behaviors and actions that manifest during therapeutic encounters. A key nurse contributor cited was behaviors and actions that portray poor reception and hospitality and not expressing concern at earlier stages of therapeutic encounter. Poor reception and hospitality were said to be characterized by a tendency of nurses not greeting the clients or responding to greetings from a client and self-pride. These behaviors and actions were said to negatively impact how the relationship could be constructed when a nurse encounters a client. Another dominant nurse contributor to poor relationship was poor communication. Poor communication was said to be characterized by a nurse using bad and harsh language, speaking in anger, shouting and verbal reprimands towards clients, not listening to clients, not making eye contacts when speaking, not being able to speak local language, failure to offer adequate information about medical interventions and side effects and overall negative attitudes. These factors were cited to limit client freedom and engagement in care and decision making consequently constructing negative relationships. Other nurse contributors of poor nurse-client relationships included: poor quality of services, poor relationship among peers, job dissatisfaction and unstable mental health. Job dissatisfaction and unstable mental health were cited to reduce work morale and commitment resulting into poor quality of care and negative therapeutic interactions. Some participants commented:

*“What contribute to poor relationship between a client and a nurse is self-pride among nurses. You find a nurse who is too proud of herself. She does not respond to greetings, or she does not greet clients…she is just busy chatting without showing concern about client’s problems. She does not listen, she does not care and is not attentive when speaking to her” (Client, Dispensary)*

*For example, if a child receives vaccination, she may develop fever and we as clients do not know that this is normal…that the child may not breastfeed well after vaccination or may cry excessively. We are not told that we should not worry because when you reach the facility they just inject ‘chwi…chwi…chwi’ and tell you to go home. You reach home and the child is not breastfeeding or develops fever, and you have to go back to the hospital. This increases the cost, and you reach the hospital they tell you that this is normal. Something which the nurse should have told me before instead going back the second time. Therefore, the relationship becomes poor because the nurse did not give me adequate education about side effects of vaccine (Client, Health Centre)*

#### Client contributors of poor nurse-client relationships

Clients were also cited by many participants to contribute to poor relationships.The client contributors to poor nurse-client relationship were mainly related to being ‘much know’, late attendance, failure to adhere to established procedures at the facility, negative attitudes towards nurses and poor communication*.* In comparison to nurses (above), poor communication among clients for instance, were characterized by a tendency of being troublesome/ with bad language towards nurses, having self-pride and disrespect towards nurses, portraying anger when explaining problems and being naturally non-civilized and angry because of cultural upbringing from childhood. These behaviors were cited to sour interactions between clients and nurses constructing negative relationships. Other client contributors cited were inadequate education and poor awareness about healthcare, poverty which creates inability of the client to meet the cost of care, hurrying or forcing to receive certain services that they do not deserve, overall dissatisfaction with care, faith in traditional healers which creates negativity towards formal healthcare providers and unstable mental health. These factors were said to created frequent frictions, conflicts and blames that translates to poor nurse-client relationships. Moreover, unstable mental health for both nurses and clients were cited to negatively impact the quality of interactions that is critical in building a stable therapeutic relationship. Some of the participants commented:

*Most of the clients nowadays knows everything. We call them ‘Bishololo’ because they know everything, and you ask yourself why did they come to the hospital when they appear to know everything. They know which medications they need…’eeh write me Amoxicillin caps…write me that’ so it is like they teach a nurse to do her work. You may be inserting an IV drip and they keep instructing you where and how to insert…this contributes to many conflicts especially in urban areas because rural people are much calmer…they do not know many things, but this (not knowing many things) may also contribute to poor relationship when they miss certain services (MCH administrator)*

*Most of us are suffering from mental health diseases. Both nurses and clients are suffering…we are facing many stresses of life and no support… this contributes to poor relationships (Client, Hospital)*

#### Health system contributors of poor nurse-client relationships

A number of health system contributors to poor nurse-clients relationships emerged in participants’ accounts. Most of these contributors were related to inadequate resources that create conflicts and frictions between clients and nurses as well as ineffective procedures for gathering and handling client’s dissatisfactions and conflicts with nurses. Specifically, healthcare system contributors ranged from inadequate resources (inadequate number of nurses, inadequate medicines and medical supplies, infrastructure, and guidelines) in relation to client load. Another dominant contributor was concerns of poor human resource for health management practices including mistreatment of staff by leaders, inadequate financial incentives, small and delayed salaries, and promotions. Inadequate salaries, incentives and work environments were cited to create dissatisfactions among nurses which may translate to poor quality of care and poor inter-personal communication with clients. Likewise, inadequate resources in healthcare facilities for instance, were cited to fuel misunderstandings and conflicts between nurses and clients resulting into poor relationships. There were also concerns of overall lack of client trust towards public healthcare facilities and providers due to persistent malpractices and negative client experiences when seeking MCH care. Additionally, inadequate policy implementation was cited to contribute to inadequate resources and malpractices that fuel negative therapeutic interactions in MCH Care. Finally, there was a concern of absence of an independent department or agency specifically responsible for gathering, analyzing and communicating clients and providers’ complaints. Considering inadequate resources as a contributor of poor nurse-client relationships, one participant detailed how the absence of PoP (Plaster of Paris)- a medical supply commonly used for injuries- resulted into poor nurse-client relationship:

*My child had an injury, and we went to the facility and the doctor wrote a prescription and when we went to the nurse to give us the materials, she told us that they are not available. My husband became so angry and furious that he slapped the nurse … asking that why a big hospital like that has no POP materials…It was a big conflict, and I even became afraid. Yes, patient themselves often contribute to poor relationship with nurses (Client, Health center)*

## Factors shaping good nurse-client relationship

Participants were also asked about the contributors of good nurse-client relationships. Looking across transcripts, contributors of good nurse-client relationships were mainly the opposite of contributors of poor nurse-client relationships. For instance, nurse and clients’ contributors were mainly related to their *good behaviors and actions* that transpire *during physical therapeutic interactions* within MCH clinics and *good outcomes after such interactions*. Good behaviors, attitudes, and actions of nurses cited to contribute to good relationships included those related to good reception of the client, expressing care, good communication, better services, ongoing support, friendship and trust, and positive reputation within the community. During therapeutic interactions, *good reception by a nurse* was said to be characterized by exchanging greetings, introducing oneself, addressing worries, offering hope, asking what the problem is and promoting equality. Furthermore, *expression of care* was said to be characterized by nurse’s willingness and readiness to help, promoting clients’ rights, closeness to clients, showing interest in clients’ concerns and cooperation. Moreover, *good communication* was said to be characterized by nurses’ good and soft language, positive body language, calmness, avoidance of harshness and harassment, listening, consensus building/shared decision making and being able to speak a local language. Likewise, *better healthcare services* were characterized by nurses’ timeliness of care, correct treatment, good counseling and post-care instructions, and friendly services. After therapeutic interactions, nurses’ confidentiality of patient information, continued friendship and continued communication and support surfaced as shaping good nurse-client relationship. These good nurses’ behaviors and actions were cited to increase client’s satisfaction with both interpersonal and technical quality of care consequently constructing good therapeutic relationships.

Although nurses’ positive behaviors and actions dominated as shaping good relationship, clients’ good behaviors, attitudes and actions were also mentioned. For instance, during therapeutic interactions, clients’ timely arrival to the clinic, client’s positive attitude and trust towards nurses, adherence to instructions, good communication, openness and understanding what services they deserve, and thankfulness were cited as shaping good nurse-client relationship. After the interaction, clients’ satisfaction with care/absence of complaints, friendship beyond MCH clinics often accompanied with gifts and promoting goodness of nurses in the community were cited as shaping good nurse-client relationship. Some participants commented:

*[Good relationship] occurs when you receive the patient well, how you introduce yourself and offer medical services. At the end of the day, you build friendship, you stay connected and she may call you if she has a problem. It occurs when the client becomes satisfied with your services, and you may become like family friends. She may even bring you gifts because you offered good care (Nurse, Health Centre)*

*What I know is that a good relationship between nurses and clients is shaped by three things. First is trust between client and nurse, meaning a client has high trust towards a nurse. Second, confidentiality meaning nurse keeps patient information confidential. Third, willingness and readiness of a nurse to leave all other things to offer care to the client, hospitality and treating the client like a king. If are done, there will always be good relationship between a nurse and client” (Client, Dispensary)*

### Suggestions for improving nurse-client relationship

A range of suggestions were mentioned for strengthening nurse-client relationships. Similar to descriptions of nurse, clients, and health system contributors of poor nurse-clients relationship, the suggestions for improvements included those focusing on nurses, clients, and health system. Of note, most of the recommended strategies are those focusing on health system. Suggestions focusing on nurses included those related to improving their practices and satisfactions such as: awards and recognition of good nurses, continued professional development, peer to peer learning and mentorship including pairing good and experienced nurses with bad and inexperienced nurses in service delivery, learning local language (Sukuma) for easy communication and insisting on personal devotion to nursing professional and work ethics in different platforms. These strategies were described to boost work morale among nurses, build confidence and expertise needed to offer quality MCH care, build a caring heart and closeness with clients that could construct good therapeutic interactions and satisfaction among clients. Furthermore, the suggestions focusing on clients included: education and community sensitization and insisting on good behaviors and attitudes towards nurses on different platforms. These suggestions were described to possibly fuel positive interactions between clients and nurses in MCH care points. Finally, suggestions focusing on health system included: improving service quality and working conditions, improving complaints mechanisms, increasing and better management of nursing workforce, reducing politicization of healthcare services, improving remuneration and incentives, extended mentorship and, strengthening screening of students seeking to join nursing schools and improving nursing curriculum to generate graduates with self-drive and good relationship with clients. These suggestions were said to have the potential to reduce tensions among clients and nurses within therapeutic MCH settings. Table 3 summarizes these strategies. However, some participants commented:

*Experienced nurses need to support less experienced ones. If possible, leaders should pair nurses with bad reputation and less experience with those with good reputation and more experiences for them to gain desired competences which are needed to reduce conflicts with clients (MCH stakeholder)*

*A major strategy is continued community education so that our clients become aware of the importance of coming early to the clinic. Nurses need to be trained on customer care skills, on how to give health education and counsel clients effectively (Nurse, Dispensary)*

*Nurses need to lean local language for the to communicate smoothly with clients…. At least few words (Client, Dispensary)*

*Politics and health are different professions. If you are a politician talk politics and leave health issues to health experts. Politicians tell lies to our clients. People are given empty promises by politicians just to find that they have to purchase some things when they come to hospitals. Politicians need to let health experts do their work while they focus on politics…this will reduce conflicts in healthcare settings (Nurse, Health Centre)*

**Table 3:** Suggestions for strengthening nurse-client relationships

**Strategies focusing on nurses**	**Strategies focusing on Clients**
**Awards and recognition of good nurses** **Continued professional development on:** • ***Customer care skills*** • ***Skills for improving nurse-client relationship*** • ***Time management skills*** • ***Communication skills*** • ***Nursing service delivery competences*** • ***Respective nursing care skills*** • ***Counseling skills*** • ***Delivery of health education session for low literacy clients*** • ***Develop a habit of updating one’s nursing skills*** • ***Basic clients’ rights in MCH care*** • ***Induction course for new employees on nurse- client relationships***	**Education and community sensitization on:** • *The importance of early healthcare seeking* • *The type of services they deserve* • *Swahili language (in rural areas) or coming with interpreters* • *Communication skills in healthcare settings* • *Reputable sources of information* • *Basic clients’ rights within healthcare settings*
	**Insisting on the followings on different platforms** • *The importance of coming early to the clinic (within scheduled time)* • *Reducing anger in order to receive good care* • *Being respectful and thankful to nurse’s efforts* • *Using good language and using civilized communication* • *Reliance on reputable health information sources* • *Adherence to instructions from nurses*
**Promoting peer to peer learning and mentorship** • ***Training nurses as peer mentors on nurse-client relationship*** • ***Pairing good and/or experienced nurses with bad or junior nurses when planning for working shifts*** • ***Nurse to develop a habit of sharing/giving feedback to peers what they have learnt in seminars and workshops*** • ***Learning from best performing private sectors*** • ***Improving relationship and cooperation among nurses***	
**Insisting on personal devotion to nursing professional in different platforms on these aspects:** • ***Valuing nursing work*** • ***Reminding nurses to fulfil their responsibilities, adherence to nursing ethics and having a nursing call*** • ***Respect of clients’ views*** • ***Respect of client’s rights including choice of a nurse to receive care from*** • ***Willingness to receive feedback and improve oneself*** • ***Learning local language (Sukuma)***	
**Others** • ***Peer policing- a tendency of nurses to monitor and warn fellow nurses*** • ***Linking nurses’ performance evaluation to renumeration*** • ***Nursing leaders to fulfil their responsibilities***	
**Strategies focusing on health facilities/health sector**	
**Improving quality of services and working conditions** • ***Improving availability of medicine and medical equipment*** • ***Improving friendliness of working environment*** • ***Improving MCH infrastructure*** • ***Ensuring availability of medicine and medical supplies for exemption groups*** • ***Establishing health service grades (based on ability to pay) without compromising the basic quality of care*** • ***Expanding formal healthcare service options in rural areas to promote patient choice***	
**Increasing and better management of nursing workforce** • ***Employing more nurses*** • ***Increasing facility income to generate funds needed to recruit more nurses*** • ***Ensuring nurses are paid as per their job contracts*** • ***Employing volunteers’ nurses to cover for deficits*** • ***Ensuring nurses work within hours stipulated in labor laws***	
**Feedback and punishments** • ***Leaders to offer feedback to nurses on their quality of services*** • ***Demotion of nurses with bad reputation or moving them to rural areas***	
**Improving renumeration and incentives for nurses** • ***Improving salary of nurses/ salary increments*** • ***Timely salary and oncall/overtime payments*** • ***Offering financial incentives for nurses to reach clients at different levels*** • ***Financial motivation to those performing well***	
**Extended Mentorship** • ***CHMT to frequently visit facilities for extended onsite mentorship (e.g., full day mentorship)*** • ***The Ministry of health to visit facilities for mentorship and developing solutions to existing challenges*** • ***Establishment of a specific agency/ focal persons for continued capacity building and mentorship of nurses on provider–client relationships***	
**Reducing politicization of medicine** • ***Politicians to avoid making unrealistic healthcare commitment and promises to people*** • ***Politicians to avoid interference with nurses’ work***	
**Debates, conferences, seminars and workshops** • ***Holding debates among nurses to identify challenges they face*** • ***Meetings bringing together nurses and clients to identify challenges in their relationship and generating joint solutions*** • ***Conferences, seminars and workshops of nurses at different levels (e.g., districts and regions) to sensitize and remind nurses about nursing ethics and nursing care***	
**Improving complaints mechanisms** • ***Establishment of independent system or agency for tracking, gathering, analyzing and publishing clients and nurses’ complaints. The agency could share with responsible authorities, track implementation and give feedback to people who made the complaints*** • ***Giving timely feedback to clients about their complaints and suggestions***	
**Nursing school student screening and curriculum** • ***Adequate screening of students joining nursing schools to maximize enrollment of those with nursing call*** • ***Strenghethern nursing school curriculum by including topics on nurse-client relationships and patient centered care*** • ***Ensure availability of trainers of nurse-client relationships in nursing schools***	
**Improving efficiency of the Nursing Professional Council** • ***Nursing council to develop and implement customer care training programs (seminars and workshops)*** • ***Nursing councils to remind nurses their work ethics, take actions to those with no work ethics and congratulating (letters) those with better performance on work ethics***	
**Community education and sensitization** • ***Use of TVs and Radio to offer health education to the community*** • ***Educating community on early health care seeking and male engagement, and adherence to medical advice and medications*** • ***Educating community on service delivery process*** • ***Engagement of CHWs in community sensitization and education*** • ***Educating community on clients’ rights***	
**MCH leaders to be responsible** • ***MCH leaders to lead by example i.e., participate in service delivery not just sitting in offices*** • ***Leaders to channel nurses’ concerns to the MoH, make follow up and give feedback to nurses*** • ***Leaders to follow up closely and timely on requests for medicine and medical supplies***	
**Others** • ***Establishing a system to monitor, control and regulate the quality of health information in internet platforms and social media*** • ***Establishing a public relations department/focal person in every facility and charge it with community education and sensitization*** • ***Reducing the cost of care*** • ***More research on how to strengthen nurse -client relationships*** • ***Making use of the findings of this research for improvement*** • ***Stakeholder (clients and nurses) engagement in developing health service delivery policies*** • ***Ensure adequate implementation of existing policies to create positive image of health sector among community members*** • ***Improving cooperation between the facility and local governance in surrounding communities***	

## Discussion

This study was conducted as the first step of the ongoing HCD intervention to co-design an intervention package for improving nurse-client relationships in MCH care in rural Tanzania. Ideally, to address the contributors of poor nurse-client relationship, a consideration of an innovative approach that brings nurses and MCH clients together as partners in the intervention design and evaluation process need to be made. It is for this reason, HCD approach is being embraced because it is considered to facilitate improvements in client, provider, and community satisfaction and increased efficiency and collaboration in public health intervention development and implementation process [27–32]. Furthermore, HCD is considered to result into more successful and sustainable interventions when compared to traditional problem-solving approaches in health care and public health in general [27–32]. Melles, Albayrak and Goossens [32] recently proposed that the implementation of HCD in healthcare need to focus on developing an understanding of the people facing a particular challenge and their needs and engaging the stakeholders from early on and throughout the design process and embracing a system-wide approach by considering interactions of factors at different levels and harmonizing individual interests to form collective interests when developing solutions to the identified challenges. Consequently, we first sought to generate an understanding of the factors shaping poor nurse-client relationships to guide future steps of HCD process. Therefore, the study was conducted with an overarching question, ‘what are the contributors of poor nurse-client relationship in MCH care that could form the basis for co-designing effective interventions?'

Our findings unmasked a range of factors shaping poor nurse-client relationships in MCH care. Nurses’ behaviors, attitudes and actions that portray poor reception and hospitality upon client’s arrival to the facility constructs poor relationships. Likewise, nurses’ failure to express care and concerns towards the clients and their suffering, poor communication in terms of abusive, and unfriendly language, and even the harsh tone, negativity towards clients and poor services dominated as the main drivers of poor relationship. However, expressing positive behaviors, attitudes and actions emerged to fuel positive relationships. At the heart of poor behaviors, attitudes and actions was the concern that nurses’ dissatisfaction with working conditions, remuneration and poor motivation as well as associated mental instability impacts how they construct relationships with their clients. Most of these findings are not novel. A large body of literature have always linked poor provider–client relationship with providers’ behaviors, attitudes, and actions in Zimbabwe [13], Malawi [12, 20], Tanzania [14, 17, 23], Kenya [16], Iran [18], Spain [19], Australia [36] and Ghana [37]. For instance, a systematic review of 81 articles, 68% of which were from Africa by Mannava et al. [20] indicated that positive nurses’ behaviors including expression of care, respect, sympathy and helpfulness construct good relationship while negative attitudes including verbal abuse, rudeness and poor communication not only constructed poor relationship but also reduce client healthcare seeking and health outcomes. This indicates that interventions that promote positive nurses’ behaviors, attitudes and actions manifesting during therapeutic interactions in MCH forms an important partway to improving nurse -client relationships, client healthcare seeking behaviors and health outcomes. It is also important to note that similar findings have also been reported by Isangula [23] when examining trust in doctor- client relationship in a similar setting. Meaning, promotion of positive behaviors, attitudes, and actions, need to extend beyond nurses to other healthcare providers who interacts with clients across MCH service points within healthcare facilities.

While most literature have focused on providers’ behaviors, attitudes and actions that fuel poor nurse-client relationships in health care, for instance [18–23] and [36–39], there appears to be paucity of literature focusing on clients’ contributors of poor nurse-client relationships. Consequently, most of the existing interventions -whether governance instruments, competence -based or political- prioritize or lays blames to providers (nurses included) as the prime source of poor provider-relationships in therapeutics encounters [17–25]. However, the finding of this study indicate that clients have a notable contribution to poor nurse-client relationships. A tendency of clients being ‘much know’ meaning they often ‘instruct’ nurses what to do, late attendance, non-adherence to established procedures at the facility, negative attitudes towards nurses, poor communication, inadequate education and awareness, poverty, hurrying and forcing to receive certain services, dissatisfaction with care, faith in traditional healers and unstable mental health emerged as contributors. These findings point to a suggestion that attempt at improving nurse-client relationships need interventions that not only focus on nurses but also clients’ behaviors, attitudes, and actions as well as improving client’s awareness on health issues, process of care and health service purchasing power.

One issue that need close examination is a tendency of nurses citing clients as ‘*much know’*. This is complex problem and may be reflecting deeper issues of medical knowledge custodianship and the prevailing power-dynamic between nurses and clients. On the one hand, medical providers are often considered the custodians and reliable sources of ‘correct’ medical knowledge and practices. However, some research indicates clients often consider easiness of accessibility of medical information at the risk of inaccuracy and non- reliability [40–42]. A recent evidence synthesis study [41] indicated that clients “tend to prefer the Internet for the ease of access to information although they continue to trust providers because of their clinical expertise and experiences. Receiving medical information from internet sources for example may mean that nurses like other medical providers may be challenged as clients are becoming more and more empowered through other sources of medical information. This imply that empowering clients by facilitating easy access to information about medical topics should be approached with care since they may access incorrect information that could negatively impact their therapeutic interactions with medical providers in healthcare settings.

On the other hand, citing clients as ‘*much know’* may portray the prevailing power dynamics between nurses and clients. The education disparity between nurses and clients noted in this rural setting (see demographic information) may contribute to feelings of superiority or authoritarian tendency among some nurses. Therefore, some nurses may resist accommodating clients’ insights and preferences on the treatment process. This may also indicate limited understanding of the value of client participation in decision making as part of promotion of client-centered care among some nurses. It is important to note that power-dynamics and poor engagement of clients in treatment decision making has been reported as common in the study settings and other low-income healthcare settings [20–23]. Consequently, some authors encourage nurses to focus on working with the clients through power sharing and negotiation rather than being preoccupied with doing everything to them without engagement [43, 44]. Some authors recommend adapting the traditional approaches to provider–client and communication in therapeutic settings to and optimize client engagement in health decision making as a means of minimizing the negative impact of client access to health information through non-medical sources [43]. This approach is expected to result into client empowerment, partnership, client-centered care and shared decision making which positively impact healthcare seeking and outcomes [42, 44].

Literature in low- and middle-income countries suggest that availability of healthcare resources (enough providers, medicines and medical equipment) is essential in creating smooth environment where provider–client relationships can be established and sustained [23]. This partly explain why a range of health system factors emerged in this study appears to impact how nurse-client relationships are constructed within MCH care. Inadequate resources (medicines, medical supplies and equipment), infrastructures, financial resources and nursing workforce, poor human resource for health management practices, and inadequate policy implementation emerged to create frequent tensions and frictions among clients and nurses consequently impacting their relationships. Inadequate resources for instance may influence negative nurses’ behaviors and attitudes towards clients [20]. Relatedly, inadequate resources limit nurses’ capacity to utilize the full potential of her expertise and create blames and conflicts with clients who miss specific services due to lack of resources [17–23, 45]. It is important to also note that inadequate resources may create tensions between nurses and clients due to prevailing politicization of medicine where politicians often make promises of quality and free MCH care to clients contrary to what facilities could actually offer [23, 45]. A study of providers in Tanzania [45] indicated that politicians make promises of free health service to pregnant women when the healthcare facilities have limited budget to ensure availability of resources to meet these political commitments. Consequently, the nurses and other providers carry the burden and become the targets of attacks and abuse by unsatisfied/angry clients who are not happy with inadequate resources for healthcare services and are often considered corrupt [45]. This is evidenced by an incident described by one of our participants where a nurse was physically abused by the client for the healthcare facility not having the POP (see above).

Most importantly, lack of patient trust towards health facilities and healthcare providers and high faith in traditional medicine emerged to contribute to poor nurse-client relationships. While similar findings have been previously reported in a similar setting with respect to doctor- patient relationships [23], it may be that nurses’ behaviors, attitudes and actions portraying poor hospitality and non- expression of care as well as concerns of inadequate resources in formal healthcare system pushes clients to have higher trust and faith in informal practices [23]. Furthermore, conducting a study in a rural region with wealth traditional practices that appears to compete with formal western care may explain high preference to traditional healers [23]. An important finding was the absence of an independent agency for gathering, processing and sharing clients and nursing feedbacks within healthcare system. This emerged as important because of many concerns of dysfunctional suggestion box system in terms of lack of education among clients, delay in working on suggestions and lack of feedback to the people who offered complaints. Similar challenges have been detailed previously in a similar setting [23]. What these findings points to is that efforts to improve nurse client-relationship requires combination of interventions that not only seek to promote positive behaviors among nurses and clients but also ensuring availability of resources, nurse workforce management practices and building confidence and faith of community members towards formal western care.

Taken together, all these findings indicate that there may not be a single best fit solution that could be employed to strengthen nurse-client relationships. This is because such relationship is impacted by complex factors operating at the level of nurses themselves, clients, and health system. This may explain why a combination of intervention were proposed. First, those focusing on nurses such as awards and recognition, continued professional development, peer to peer learning and mentorship including pairing good and experienced nurses with bad and inexperienced nurses in service delivery and using nursing platforms (meetings, conferences, workshops etc.) to insist on personal devotion and adherence to nursing professional and work ethics. Most of these suggestions have been proposed in previous literature [17–23]. For instance, Kumbani et al. [20] proposed strengthening workforce development, including training in communication and counselling skills as a pathway to improving nurses’ behaviors and attitudes as attempt to improve maternal health services and outcomes in Malawi. Second, those focusing on clients including education and community sensitization and insisting on good behaviors and attitudes towards nurses on different platforms. Third and final, those focusing on health system including improving service quality and working conditions, increasing and better management of nursing workforce, improving remuneration and incentives, extended mentorship, improving complaints mechanisms and strengthening nursing school’s student screening and nursing curriculum. It is important to note that most of these suggestions are among the key recommendations of studies on provider–client relationships in low- and middle-income countries[36–39, 46]. Underemphasis on the role of improving provider-client relationships in health care and inadequate funding may have contributed to less implementation of most of these suggestions.

### Limitations

This study is not without limitations. First, it was conducted with the purpose of generating evidence to guide co-design of interventions using HCD approach. The focus on contributors that could shape development of intervention may have limited the scope of our findings by excluding themes and subthemes that may not be seen from an intervention design perspective. However, we believe that the findings may have broader use in nursing and MCH practice beyond the realm of design thinking process. Second, the study uses nurses as providers, to generate insights to facilitate the co-development of a prototype for strengthening interpersonal relationships in MCH care in a rural setting. However, patients interact with a multidisciplinary team of providers across different service points within healthcare settings. Conducting a similar study with other providers such as doctors, clinicians, allied workers, and, in a different clinical setting may yield different results. However, this being the first study employing HCD approach in this rural context, a belief is that if deemed successful, the emerging prototype could be tested/applied in diverse clinical settings. Future inquiries may extend beyond nursing profession and rural contexts.

## Conclusions

The factors shaping poor nurse- client relationships appear to extend beyond nurses to both patients and healthcare system and facilities. These results may inform the design of new initiatives and the policies that support them in order to strengthen interpersonal relationships in health care settings more broadly. Therefore, implementation of effective interventions for addressing identified factors considering feasibility and acceptance to both nurses and clients using novel strategies such as HCD could pave the way for employing good nurse-client relationships as a tool for improving performance indicators and health outcomes within MCH care.

## Supplementary Information

**Additional file 1.**

**Additional file 2.**

## Data Availability

The data that support the findings of this study are available from the School of Nursing and Midwifery at the Aga Khan University but restrictions apply to the availability of these data under the current study, and so are not publicly available. Data are however available from the corresponding author upon reasonable request and with permission of the School of Nursing and Midwifery at the Aga Khan University.

## Abbreviations

FGD: Focus Group Discussions
HCD: Human Centered Design
KII: Key Informant Interviews
MCH: Maternal and Child Health
NatHREC: National Health Research Ethics Sub-Committee
NIMR: National Institute for Medical Research
