# Supplementary material for: Factors shaping good and poor nurse-client relationships in maternal and child care: a qualitative study in rural Tanzania
Source: BMC Nurs. 2022 Sep 5;21:247. doi: 10.1186/s12912-022-01021-x (PMC9443654; doi:10.1186/s12912-022-01021-x)
Supplement: Supplementary file 1 — Additional file 1. [file 12912_2022_1021_MOESM1_ESM.docx]

**INTERVIEW GUIDES**

**FOCUS GROUP DICUSSION GUIDE WITH NURSES AND CLIENTS**

1. What does a good nurse-client relationship mean to you? (*Probe: ever experienced good relationship with nurse/clients? What happened?)*
2. What does a bad/poor nurse-client relationship mean to you? (*Probe: ever experienced bad relationship with nurse/clients? What happened?)*
3. What are the consequences of having a bad relationship with your nurse/client in MCH care? *(Probe:* *consequence to nurses, consequence to clients, consequence to health system?*
4. What are the contributors to a bad relationship between nurses and their clients in MCH care in Shinyanga? *(Probe: Nurse factors, client factors, health system factors?)*
5. What are your recommendations (strategies) for strengthening relationship between nurse and their clients within MCH care in Shinyanga? *(Probe: How each strategy mentioned can be made much more successful? Key considerations when implementing each strategy? What are the possible barriers that can impact the successful implementation of each strategy mentioned?)*
6. Any other comment in relation to nurse-client relationship in Shinyanga?

**KII GUIDE WITH MCH ADMINISTRATORS**

1. What are your roles and responsibilities in relation to MCH care?
2. What does a good nurse-client relationship mean to you? (*Probe: ever received clients complements of nurses? What happened?)*
3. What does a bad/poor nurse-client relationship mean to you? (*Probe: ever handled client’s complaints about nurses? What happened?)*
4. What are the benefits of having a good relationship with your nurse/client in MCH care?
5. How a bad relationship between nurses and clients have impacted MCH care in Shinyanga? *(Probe: consequence to nurses, consequence to clients, consequence to health system?*
6. What are the contributors to a bad relationship between nurses and their clients in MCH care in Shinyanga? *(Probe: Nurse factors, client factors, health system factors?)*
7. What are the existing strategies employed for strengthening nurse-client relationships in MCH care in Shinyanga?
8. What are your recommendations (strategies) for strengthening relationship between nurse and their clients within MCH care in Shinyanga? *(Probe: How each strategy mentioned can be made much more successful? Key considerations when implementing each strategy? What are the possible barriers that can impact the successful implementation of each strategy mentioned?)*
9. Any other comment in relation to nurse-client relationship in Shinyanga?
