# Supplementary material for: Factors shaping good and poor nurse-client relationships in maternal and child care: a qualitative study in rural Tanzania
Source: BMC Nurs. 2022 Sep 5;21:247. doi: 10.1186/s12912-022-01021-x (PMC9443654; doi:10.1186/s12912-022-01021-x)
Supplement: Supplementary file 2 — Additional file 2. [file 12912_2022_1021_MOESM2_ESM.docx]

**Individual Participants demographics**

| **Hospital** | 20yr, Female, Married, 1 Child, Form III, Wami  22yr, Female, Married, 2 Children, STD VII, Mwamala  22yr, Female, Married, 2 Children, STD VII, Bulambila  35yr, Female, Married, 4 Children, STD VII, Hosp area  20yr, Female, Single, 1 Child, Form IV, Hosp area  19yr, Female, Married, 1 Children, Form II, Hosp area  34yr, Female, Married, 5 Children, STD VII, Hosp area  33yr, Female, Married, 5 Children, STD VII, Hosp area | 31yr. Female, Married, Diploma, 4 years in service  53yr, Female, Married, Diploma, 15 years in service  45yr, Female, Married, Diploma, 21 years in service  31yr, Female, Married, Certificate, 8 years in service  25yr, Female, Married, Diploma, 1 years in service  31yr, Female, Married, Diploma, 7 years in service  52yr, Female, Married, Certificate, 19 years in service  32yr, Female, Married, Diploma, 11 years in service |
| --- | --- | --- |
|  |  | 26yr, Male, Single, Diploma  38yr, Female, Single, Certificate  50yr, Female, Married, Certificate  50yr, Female, Married, Diploma  24yr, Male, Single, Diploma  26yr, Male, Married, Diploma  32yr, Female, Married, Certificate |
| Health Centre | 25yr, Female, Married, 1 Child, STD VII, Kitangili,  28yr, Female, Married, 1 Child, Form IV, Majengo mapya,  30yr, Female, Married, 3 Children, STD VII, Ndembezi  26yr, Female, Married, 1 Child, University, Uzunguni,  26yr, Female, Married, 1 Child, Bachelor, Mwalugoye,  37yr, Female, Married, 3 Children, University, Majengo | 38yr, Female, Married, Diploma  44yr, Female, Married, Diploma,  32yr, Female, Married, Certificate  33yr, Female, Married, Diploma  34yr, Female, Married, Diploma  44yr, Female, Married, Diploma  35yr, Female, Married, Diploma  36yr, Female, Married, Diploma |
| Dispensary | 25yr, Female, Married, 1 Child, FORM IV, Ndala  27yr, Female, Married, 2 Child, Form IV, Ndala  30yr, Female, Married, 3 Children, STD VII, Ndala  23yr, Female, Married, 2 Child, STD VII, Ndala  38yr, Female, Married, 2 Child, Form IV, Ndala  18yr, Female, Single, 0 Children, Ndala,  29yr, Female, Single, Degree, no child, Ndala  16yr, female, Single, Diploma, no children, Ndala | 50yr, Female, Married, STD VII,  42yr, Female, Married, Form IV,  25yr, Female, Married, Form IV  33yr, Male, Married, Form IV,  44yr, Female, Married, Form IV,  23yr, Female, Married, Form IV,  31yr, Female, Married, Form IV |
|  | 36yr, Female, Married, 3 Children, STD VII, Nheregani  32yr, Female, Single, 5 Children, Form IV, Nheregani  19yr, Female, Single, 2 Children, STD VII, Nheregani  19yr, Female, Single, 3 Children, Form IV, Nheregani  35yr, Married, 0 Child, STD VII, Nheregani  35yr, Married, 5 Children, STD VII, Nheregani,  22yr, Married, 2 Children, STD VII, Nheregani  34yr, Single, 2 Children, STD VII, Nheregani |  |
|  | 31yr, Female, Married, 5 Children, Mwawaza  29yr, Female, Married, 3 Children, STD VII, Mwawaza  51yr, Female, Married, 9 Children, Mwawaza  28yr, Female, Married, 3 Children, Mwawaza  20yr, Female, Married 1, Mwawaza  29yr, Female, Single, 3 Children, Mwawaza |  |
| **KEY INROMANT INTERVIEWS** | | |
| HFGCC | 50Yrs, Male, 2 years of leadership, Form IV, Married |  |
| Health Admin | 46, Male, Married, Master’s Degree, 10 years of Leadership |  |
| Facility Incharge | 47, Female, Married, Diploma, 10 years of leadership, Dispensary |  |
| Facility Incharge | 36, Male, Married, Bachelor Degree, 5 years of leadership, Hospital |  |
| Matron | 48, Female, Married, Bachelor of Nursing, 3 years of Leadership, Hospital |  |
| Matron, | 48, Female, Married, Bachelor of Nursing, 15 years of leadership, health centre |  |
| Health Admin | 42, Male, Married, MD, 10 years of leadership |  |
| Health Admin | 44, Female, Married, Bachelor of science in nursing, 14 years of leadership |  |
| MCH coordinator | 36, Single, Bachelor of nursing, 3 years of leadership, Health Centre |  |
| MCH Admin | 39, Female, Married, Advanced Diploma, 9 years of leadership, |  |
| MCH Admin | 48, Female, Bachelor of nursing, 10 years of leadership |  |
